# Housing Status, Cancer Care, and Associated Outcomes Among US Veterans

**DOI:** 10.1001/jamanetworkopen.2023.49143

**Published:** 2023-12-21

**Authors:** Hannah C. Decker, Laura A. Graham, Ashley Titan, Hemal K. Kanzaria, Mary T. Hawn, Margot Kushel, Elizabeth Wick

**Affiliations:** 1 Department of Surgery, University of California, San Francisco; 2 Health Economics Resource Center, Veterans Affairs Palo Alto Health Care System, Menlo Park, California; 3 S-SPIRE, Stanford University, Stanford, California; 4 Department of Surgery, Stanford University, Stanford, California; 5 Department of Emergency Medicine, University of California, San Francisco; 6 Benioff Homelessness and Housing Initiative, University of California, San Francisco; 7 Division of General Internal Medicine, Center for Vulnerable Populations, Zuckerberg San Francisco General Hospital and Trauma Center, University of California, San Francisco

## Abstract

**Importance:**

Cancer is a leading cause of death among older people experiencing homelessness. However, the association of housing status with cancer outcomes is not well described.

**Objective:**

To characterize the diagnosis, treatment, surgical outcomes, and mortality by housing status of patients who receive care from the US Department of Veterans Affairs (VA) health system for colorectal, breast, or lung cancer.

**Design, Setting, and Participants:**

This retrospective cohort study identified all US veterans diagnosed with lung, colorectal, or breast cancer who received VA care between October 1, 2011, and September 30, 2020. Data analysis was performed from February 13 to May 9, 2023.

**Exposures:**

Veterans were classified as experiencing homelessness if they had any indicators of homelessness in outpatient visits, clinic reminders, diagnosis codes, or the Homeless Operations Management Evaluation System in the 12 months preceding diagnosis, with no subsequent evidence of stable housing.

**Main Outcomes and Measures:**

The major outcomes, by cancer type, were as follows: (1) treatment course (eg, stage at diagnosis, time to treatment initiation), (2) surgical outcomes (eg, length of stay, major complications), (3) overall survival by cancer type, and (4) hazard ratios for overall survival in a model adjusted for age at diagnosis, sex, stage at diagnosis, race, ethnicity, marital status, facility location, and comorbidities.

**Results:**

This study included 109 485 veterans, with a mean (SD) age of 68.5 (9.7) years. Men comprised 92% of the cohort. In terms of race and ethnicity, 18% of veterans were Black, 4% were Hispanic, and 79% were White. A total of 68% of participants had lung cancer, 26% had colorectal cancer, and 6% had breast cancer. There were 5356 veterans (5%) experiencing homelessness, and these individuals more commonly presented with stage IV colorectal cancer than veterans with housing (22% vs 19%; *P* = .02). Patients experiencing homelessness had longer postoperative lengths of stay for all cancer types, but no differences in other treatment or surgical outcomes were observed. These patients also demonstrated higher rates of all-cause mortality 3 months after diagnosis for lung and colorectal cancers, with adjusted hazard ratios of 1.1 (95% CI, 1.1-1.2) and 1.3 (95% CI, 1.2-1.4) (both *P* < .001), respectively.

**Conclusions and Relevance:**

In this large retrospective study of US veterans with cancer, homelessness was associated with later stages at diagnosis for colorectal cancer. Differences in lung and colorectal cancer survival between patients with housing and those experiencing homelessness were present but smaller than observed in other settings. These findings suggest that there may be important systems in the VA that could inform policy to improve oncologic outcomes for patients experiencing homelessness.

## Key Points

**Question:**

What is the association between housing status and cancer outcomes among patients treated in the US Department of Veterans Affairs (VA) health system?

**Findings:**

In this cohort study of 109 485 veterans diagnosed with lung, colorectal, or breast cancer who received VA care between 2011 and 2020, homelessness was associated with increased rates of all-cause mortality for lung and colorectal cancers.

**Meaning:**

These findings suggest that differences in survival based on housing status exist, but VA health systems may offer insights to improve oncologic outcomes for individuals experiencing homelessness elsewhere.

## Teaser

This cohort study examines the association between housing status and outcomes among veterans receiving care from the US Department of Veterans Affairs health system for colorectal, breast, or lung cancer.

## Introduction

Individuals experiencing homelessness have worse overall health status relative to the general population owing to a higher burden of chronic health conditions, increased rates of injuries, and markedly increased risk of premature mortality.^1^ As this population ages, there will be an increased need to provide care for chronic medical problems, including cancer.^1,2^ Currently, approximately 50% of single adults experiencing homelessness are aged older than 50 years, the age at which the incidence of cancer increases and many adults are recommended to undergo cancer screening.^3,4,5^ Prior studies have highlighted cancer as a leading cause of death in patients experiencing homelessness who are aged older than 50 years.^6,7^

Research evaluating the consequences of housing status on cancer treatment has focused on screening. These analyses reported that rates of age-appropriate screening for malignant neoplasms are substantially lower for people experiencing homelessness compared with the general population.^8,9,10,11,12,13^

Cancer outcomes, including stage at diagnosis, surgical outcomes, and mortality rates after diagnosis, are understudied in patients experiencing homelessness. One study examined 361 cancers among adults experiencing homelessness from a single US center and found that colorectal, breast, and oropharyngeal cancers were diagnosed at more advanced stages in these individuals and that they had higher rates of cancer mortality.^14^ To expand our understanding of oncologic care, we examined data from the US Department of Veterans Affairs (VA) health system to characterize the diagnosis, treatment, surgical outcomes, and mortality of patients receiving VA care for lung, colorectal, or breast cancer who were experiencing homelessness compared with individuals with housing. We hypothesized that veterans experiencing homelessness would have later stages at diagnosis, less overall cancer-directed therapy, poorer surgical outcomes, and higher rates of mortality than veterans with housing. The VA offers the opportunity to address these questions because it has a robust data infrastructure and has recorded housing status and addressed homelessness among beneficiaries for over a decade.^15^

## Methods

### Overall Design

This retrospective cohort study used a national sample from the VA to assess whether housing status may be associated with stage at diagnosis, treatment, and outcomes for lung, colorectal, and breast cancers. The Stanford Institutional Review Board reviewed and approved this study and its protocol, with a waiver of informed consent because of minimal risk to patients. The study followed the Strengthening the Reporting of Observational Studies in Epidemiology (STROBE) reporting guideline.

### Study Population

We used the VA Corporate Data Warehouse (CDW) Oncology Domain, which abstracts data from local tumor registries, to identify all veterans with a diagnosis of lung, colorectal, or breast cancer who received VA care between October 1, 2011, through September 30, 2020. The CDW Oncology Domain is the predecessor of the VA Central Cancer Registry, ensuring complete documentation of cancer burden in the VA. We linked Veterans Affairs Surgical Quality Improvement Program (VASQIP) data to examine surgical outcomes and complications. We focused on lung, colorectal, and breast cancers because these have clear screening guidelines, are common, and have surgery as a key component of treatment.

### Exposure

We classified veterans as experiencing homelessness if they had any indicators of homelessness in outpatient visits, clinic reminders, diagnosis codes, or the Homeless Operations Management Evaluation System^15,16^ (eAppendix in Supplement 1) in the 12 months preceding diagnosis with no subsequent evidence of stable housing (before diagnosis). Use of these multiple sources has been shown to identify more veterans experiencing homelessness than relying on any one indicator; as such, it is considered best practice to use all of the indicators to fully capture homelessness.^16^ After diagnosis, we characterized patients as persistently experiencing homelessness if they had no evidence of stable housing in the 12 months after diagnosis. We characterized patients experiencing homelessness as having gained housing if they subsequently had evidence of stable housing in the 12 months after diagnosis. We defined stable housing in this context if a patient responded that they had stable housing on the Health Factors Survey, which is administered in both outpatient and inpatient settings.

### Measurements

#### Diagnosis, Treatment Path, and Cancer Outcomes

We obtained information from the VA CDW Oncology Domain on diagnosis date, cancer type, follow-up status, presentation at a cancer conference, chemotherapy type, receipt of surgery, radiation type, duration of radiation treatment, and receipt of palliative care. We obtained information on days from diagnosis to the start of chemotherapy, to the most definitive surgery, and to the start of radiation.

#### Mortality and Censoring

We obtained mortality data from the CDW Oncology Domain and the VA Vital Status file. We defined survival time as the interval between the initial diagnosis and death from any cause. We censored veterans at the time they were documented as lost to follow-up in the CDW Oncology Domain or on October 1, 2022, when we accessed the data. We also censored veterans who changed housing status in the 12 months after diagnosis at the time of housing status change.

#### Surgical Management

We queried the VASQIP database to ascertain information on definitive cancer surgeries. The VASQIP collects data on procedures occurring at the VA only and only collects a subset of those procedures.^17^ Of the 44 138 surgeries identified in the VA Central Cancer Registry, 69% occurred at the VA and 84% of these surgeries (n = 25 737) were assessed by the VASQIP. We obtained data on emergent case status, admission status, length of stay, and postoperative complications.

#### Additional Covariates

We obtained patient-level demographic data from the CDW Patient Domain, including age, sex, race, ethnicity, and marital status. Data on veteran race (American Indian or Alaska Native, Asian, Black or African American, Native Hawaiian or Other Pacific Islander, White, or multiple races) and ethnicity (Hispanic or Latino or not) were obtained from the CDW, which is primarily based on patient self-report and has been shown to be highly accurate for assessing race and ethnicity in VA data.^18^ We assessed race and ethnicity in this study given the ways in which structural racism and discrimination reinforce poverty and contribute to homelessness. Patient-level clinical characteristics included comorbidity burden (using the Charlson Comorbidity Index), smoking status, alcohol use, and stage at diagnosis. We obtained information on the facility district where care was received.

#### Major Outcomes

The major outcomes explored were as follows: (1) treatment course, including stage at diagnosis; (2) surgical outcomes, including length of stay and major complications; (3) overall survival by cancer type; and (4) hazard ratios for overall survival in a model adjusted for measured covariates.

### Statistical Analysis

We conducted separate analyses for lung, colorectal, and breast cancers. We first performed unadjusted analysis comparing baseline characteristics and outcomes among patients with housing and those experiencing homelessness. We then developed Kaplan-Meier curves to display unadjusted mortality by cancer type. Finally, we created multivariable Cox proportional-hazard regression models to compare hazard ratios of mortality for each group for each cancer type, adjusting for age at diagnosis, sex, stage at diagnosis, race, ethnicity, marital status, facility location, and behavioral or physical health comorbidities. We divided the models into 2 distinct time periods: the first 3 months after diagnosis and beyond 3 months. We hypothesized that differences in populations with housing and individuals experiencing homelessness would differ based on time after diagnosis (with patients experiencing homelessness more likely to have adverse outcomes further out from diagnosis as follow-up and care coordination becomes more important) and because of evidence of nonproportionality in the early part of the unadjusted survival curves. We used a directed acyclic graph based on a literature review to determine the final set of covariates included in the model. Two-tailed *P* < .05 was considered statistically significant. All data analysis was performed in R, version 4.1.2 (R Foundation for Statistical Computing). Data analysis was performed from February 13 to May 9, 2023.

## Results

### Population Characteristics

There were 109 485 veterans diagnosed with cancer in our sample, with a mean (SD) age of 68.5 (9.7) years. Men comprised most of the study population (92%) compared with women (8%). A total of 1% of patients identified as American Indian or Alaska Native, 0.4% as Asian, 18% as Black or African American, 1% as Native Hawaiian or Other Pacific Islander, 79% as White, and 1% as being of multiple races. A total of 4% of veterans reported being of Hispanic ethnicity. There were 104 129 veterans (95%) with housing and 5356 (5%) experiencing homelessness at the time of cancer diagnosis. Of the cohort experiencing homelessness, 1031 (19%) gained housing within the first year after diagnosis and were censored from the survival analysis at the time of gaining housing. Breast cancer accounted for 6378 diagnoses (6%), colorectal cancer for 28 537 (26%), and lung cancer for 74 570 (68%). The group experiencing homelessness had a higher proportion of Black veterans (37% vs 17%) and was younger (mean [SD], 63.1 [8.7] vs 68.7 [9.6] years) than the group with housing. Veterans were treated at facilities across the US (Table 1 and eTable 4 in Supplement 1).

**Table 1.** Demographic Characteristics of Veterans Diagnosed With Lung, Colorectal, or Breast Cancer, Stratified by Housing Status^a^

Characteristic	Overall (N = 109 485)			Lung cancer (n = 74 570)			Colorectal cancer (n = 28 537)			Breast cancer (n = 6378)		
	With housing (n = 104 129)	Without housing (n = 5356)	*P* value	With housing (n = 71 036)	Without housing (n = 3534)	*P* value	With housing (n = 27 040)	Without housing (n = 1497)	*P* value	With housing (n = 6053)	Without housing (n = 325)	*P* value
Age, mean (SD), y	68.7 (9.6)	63.1 (8.7)	<.001	69.8 (8.3)	64.4 (7.8)	<.001	68.1 (10.9)	61.8 (9.5)	<.001	58.8 (11.7)	54.6 (9.1)	<.001
Sex												
Female	8453 (8)	495 (9)	.004	2205 (3)	153 (4)	<.001	967 (4)	55 (4)	.83	5281 (87)	287 (88)	.67
Male	95 676 (92)	4861 (91)		68 831 (97)	3381 (96)		26 073 (96)	1442 (96)		772 (13)	38 (12)	
Race^b^												
American Indian or Alaska Native	611 (1)	49 (1)	<.001	361 (1)	22 (1)	<.001	198 (1)	21 (2)	<.001	52 (1)	6 (2)	<.001
Asian	358 (0.4)	17 (0.3)		154 (0.2)	6 (0.2)		128 (1)	6 (0.4)		76 (1)	5 (2)	
Black or African American	17 036 (17)	1894 (37)		10 298 (15)	1176 (35)		5054 (20)	580 (41)		1684 (30)	138 (45)	
Native Hawaiian or Other Pacific Islander	631 (1)	34 (1)		396 (1)	22 (1)		180 (1)	9 (1)		55 (1)	3 (1)	
White	79 088 (80)	3039 (60)		55 602 (83)	2100 (63)		19 810 (78)	791 (56)		3676 (65)	148 (48)	
Multiple	687 (1)	52 (1)		440 (1)	31 (1)		172 (1)	15 (1)		75 (1)	6 (2)	
Ethnicity												
Hispanic or Latino	3521 (4)	156 (3)	.06	1715 (3)	80 (2)	.57	1513 (6)	64 (4)	.03	293 (5)	12 (4)	.36
Not Hispanic or Latino	96 912 (96)	5040 (97)		66 780 (97)	3351 (98)		24 633 (94)	1384 (96)		5499 (95)	305 (96)	
Facility district												
Continental	18 211 (17)	885 (17)	<.001	11 934 (17)	571 (16)	<.001	5114 (19)	258 (17)	<.001	1163 (19)	56 (17)	.2
Midwest	25 631 (25)	1009 (19)		18 431 (26)	717 (20)		5980 (22)	240 (16)		1220 (20)	52 (16)	
North Atlantic	23 398 (22)	1159 (22)		16 369 (23)	773 (22)		5691 (21)	309 (21)		1338 (22)	77 (24)	
Pacific	16 242 (16)	1383 (26)		10 355 (15)	863 (24)		4678 (17)	444 (30)		1209 (20)	76 (23)	
Southeast	20 647 (20)	920 (17)		13 947 (20)	610 (17)		5577 (21)	246 (16)		1123 (19)	64 (20)	
Marital status at diagnosis												
Married (including common law)	46 924 (45)	868 (16)	<.001	32 181 (45)	550 (16)	<.001	12 548 (46)	264 (18)	<.001	2195 (36)	54 (17)	<.001
Single (never married)	10 182 (10)	1205 (22)		6213 (9)	770 (22)		2946 (11)	359 (24)		1023 (17)	76 (23)	
Divorced, separated, or widowed	46 330 (44)	3240 (60)		32 254 (45)	2188 (62)		11 333 (42)	860 (57)		2743 (45)	192 (59)	
Unknown or other	692 (1)	43 (1)		388 (1)	26 (1)		213 (1)	14 (1)		91 (2)	3 (1)	
Charlson Comorbidity Index, mean (SD)	3.2 (3.1)	3.3 (3.3)	.90	3.6 (3.2)	3.7 (3.4)	.93	2.7 (3.0)	2.9 (3.2)	.43	1.3 (1.9)	1.5 (2.1)	.09
Reported tobacco use												
No	12 742 (13)	480 (9)	<.001	2431 (4)	75 (2)	<.001	7506 (29)	289 (20)	<.001	2805 (49)	116 (38)	<.001
Current	46 499 (46)	3483 (67)		37 506 (54)	2618 (76)		7650 (30)	743 (52)		1343 (23)	122 (40)	
Prior	41 480 (41)	1227 (24)		29 524 (43)	772 (22)		10 355 (41)	385 (27)		1601 (28)	70 (23)	
Reported alcohol use												
No	35 276 (38)	1326 (27)	<.001	22 536 (35)	783 (24)	<.001	9846 (40)	399 (29)	<.001	2894 (52)	144 (48)	.01
Current	37 812 (40)	2320 (47)		25 892 (41)	1551 (48)		9804 (40)	660 (48)		2116 (38)	109 (37)	
Prior	20 829 (22)	1253 (26)		15 452 (24)	894 (28)		4867 (20)	314 (23)		510 (9)	45 (15)	
No. of inpatient admissions												
0	89 051 (86)	4075 (76)	<.001	60 745 (86)	2676 (76)	<.001	22 321 (83)	1088 (73)	<.001	NA	NA	NA
1	15 012 (14)	1263 (24)		10 261 (14)	846 (24)		4683 (17)	403 (27)				
2	66 (<0.1)	18 (0.3)		30 (<0.1)	12 (0.3)		36 (0.1)	6 (0.4)				
No. of inpatient days, mean (SD)	1.8 (9.2)	6.6 (25.0)	<.001	1.7 (8.9)	6.2 (23.6)	<.001	2.3 (10.3)	8.3 (28.8)	<.001	0.3 (5.2)	2.8 (19.6)	<.001
No. of primary care visits, mean (SD)	6.2 (6.2)	6.8 (6.7)	<.001	6.5 (6.4)	7.1 (6.9)	<.001	5.7 (5.7)	6.0 (6.1)	.18	5.3 (5.7)	6.2 (6.3)	.01
No. of emergency room visits, mean (SD)	0.9 (1.5)	1.6 (2.2)	<.001	0.9 (1.5)	1.7 (2.3)	<.001	0.8 (1.4)	1.6 (2.2)	<.001	0.6 (1.2)	1.2 (1.8)	<.001

Abbreviation: NA, not applicable.

^a^ Unless indicated otherwise, data are presented as the No. (%) of patients. Percentages may not total 100 owing to rounding. eTable 4 in Supplement 1 provides additional demographic features.

^b^ Summed subset numbers may not add to category totals in the case of missing data.

### Unadjusted Analysis

Patients experiencing homelessness were notably younger at diagnosis for all 3 cancers evaluated, with a mean (SD) age at diagnosis of 64.4 (7.8) vs 69.8 (8.3) years for lung cancer, 61.8 (9.5) vs 68.1 (10.9) years for colorectal cancer, and 54.6 (9.1) vs 58.8 (11.7) years for breast cancer. For all 3 cancer types, patients experiencing homelessness were more commonly Black compared with their counterparts with housing (lung: 35% vs 15%; colorectal: 41% vs 20%; and breast: 45% vs 30%). A greater proportion of individuals experiencing homelessness were treated in the Pacific region for lung (24% vs 15%) and colorectal (30% vs 17%) cancers. For all 3 cancers evaluated, patients experiencing homelessness had similar Charlson Comorbidity Index scores compared with patients with housing, although the composition of their comorbidities differed. Patients experiencing homelessness more commonly had depression, bipolar disorder, or other mood disorders and more commonly reported tobacco use as well as alcohol use and liver disease. However, these patients less commonly reported diabetes, peripheral vascular disease, and cerebrovascular disease. These patients had more inpatient admissions, emergency department visits, and primary care visits in the year before diagnosis compared with patients with housing across cancer types (Table 1).

### Treatment Course

There were no statistically significant differences in stage at diagnosis in lung or breast cancer (Table 2) between patients by housing status. However, a higher proportion of patients experiencing homelessness presented with stage IV colorectal cancer compared with patients with housing (22% vs 19%; *P* = .02). There was no difference in treatment status for colon and lung cancers (eg, if treatment was given, no treatment was given, or active surveillance was pursued), but patients experiencing homelessness were more likely to have no treatment given in breast cancer (7% vs 4%). Similar proportions of patients in both groups were lost to follow-up in all cancer types. The type of chemotherapy given was not statistically different between groups with lung cancer, although palliative chemotherapy was more common in patients with colorectal cancer experiencing homelessness (8% vs 6%). A lower proportion of patients experiencing homelessness underwent surgery for colorectal cancer (72% vs 77%), whereas similar proportions in both groups received definitive oncologic surgery for lung and breast cancer. For those who underwent colorectal surgery, a higher proportion of patients experiencing homelessness had less than 12 lymph nodes examined in oncologic surgery compared with patients with housing (60% vs 55%). Similar proportions in both groups received palliative care in all 3 cancers studied. Greater proportions of patients experiencing homelessness and diagnosed with colorectal or lung cancer were presented at multidisciplinary cancer conferences compared with individuals with housing.

**Table 2.** Stage at Diagnosis and Treatment Course, Stratified by Veteran Housing Status^a^

Characteristic	Lung cancer (n = 74 570)			Colorectal cancer (n = 28 537)			Breast cancer (n = 6378)		
	With housing (n = 71 036)	Without housing (n = 3534)	*P* value	With housing (n = 27 040)	Without housing (n = 1497)	*P* value	With housing (n= 6053)	Without housing (n = 325)	*P* value
Cancer stage at diagnosis^b^									
0	187 (0.3)	10 (0.3)	.67	2689 (11)	134 (10)	.06	996 (18)	46 (15)	.50
I	20 722 (32)	994 (31)		6402 (27)	342 (26)		2366 (44)	126 (42)	
II	6367 (10)	303 (9)		5019 (21)	249 (19)		1344 (25)	81 (27)	
III	13 435 (21)	663 (21)		5114 (22)	295 (23)		458 (8)	31 (10)	
IV	24 500 (38)	1244 (39)		4529 (19)	283 (22)		273 (5)	15 (5)	
Treatment status									
Treatment given	55 381 (78)	2743 (78)	.41	23 965 (89)	1320 (88)	.80	5800 (96)	300 (93)	.03
Active surveillance (watchful waiting)	842 (1)	33 (1)		225 (1)	14 (1)		20 (0.3)	1 (0.3)	
No treatment given	14 749 (21)	757 (21)		2846 (11)	163 (11)		228 (4)	23 (7)	
Follow-up status									
Active	9213 (13)	442 (13)	.18	7380 (27)	354 (24)	.007	2479 (41)	129 (40)	.04
Inactive	52 058 (73)	2638 (75)		12 862 (48)	753 (50)		1697 (28)	111 (34)	
Lost to follow-up	9744 (14)	454 (13)		6797 (25)	390 (26)		1877 (31)	84 (26)	
Presentation at cancer conference									
Presented	26 134 (38)	1408 (41)	<.001	6451 (24)	362 (25)	.02	1634 (28)	84 (27)	.97
Not presented	37 899 (55)	1746 (51)		18 154 (69)	976 (67)		3719 (63)	198 (64)	
Unknown	4915 (7)	253 (7)		1751 (7)	125 (9)		504 (9)	27 (9)	
Chemotherapy type									
Adjuvant	6176 (12)	330 (12)	.20	3446 (18)	218 (20)	.001	NA	NA	NA
Concomitant or concurrent	5259 (10)	272 (10)		602 (3)	40 (4)				
Neoadjuvant	806 (2)	26 (1)		1450 (8)	98 (9)				
No chemotherapy	31 975 (61)	1585 (60)		12 285 (64)	620 (57)				
Palliative	7255 (14)	371 (14)		1228 (6)	86 (8)				
Unknown	1211 (2)	65 (3)		294 (2)	19 (2)				
Missing	18 354	885		7735	416				
Received surgery									
Surgery performed	15 896 (22)	777 (22)	.84	20 734 (77)	1083 (72)	<.001	5369 (89)	279 (86)	.21
No surgery	55 014 (77)	2751 (78)		6242 (23)	411 (27)		661 (11)	45 (14)	
Radiation									
Beam radiation	28 754 (40)	1420 (40)	.78	3252 (12)	236 (16)	<.001	2491 (41)	133 (41)	.81
None	40 896 (58)	2040 (58)		23 609 (87)	1252 (84)		3388 (56)	185 (57)	
Unknown or other	1386 (2)	74 (2)		179 (1)	9 (1)		174 (3)	7 (2)	
Duration of radiation treatment, mean (SD), d	28.6 (29.1)	29.0 (26.3)	.20	38.4 (21.7)	37.6 (13.8)	.35	41.2 (34.2)	41.1 (21.2)	.87
Received palliative care									
Palliative care	12 792 (21)	637 (21)	.55	2203 (9)	127 (9)	.63	90 (2)	5 (2)	.81
No palliative care	49 171 (79)	2383 (79)		23 447 (91)	1292 (91)		5848 (98)	310 (98)	

Abbreviation: NA, not available.

^a^ Unless indicated otherwise, data are presented as the No. (%) of patients. Percentages may not total 100 owing to rounding.

^b^ Summed subset numbers may not add to category totals in the case of missing data.

### Time to Treatment

The median time from diagnosis to chemotherapy start was similar for both patient groups for lung, colorectal, and breast cancers. The median (IQR) time to radiation was longer for patients with colorectal or breast cancer experiencing homelessness compared with patients with housing (61 [41-86] vs 55 [38-85] days and 162 [99-251] vs 135 [93-224] days, respectively), although the IQRs overlapped. The median (IQR) time to definitive surgery for lung cancer was longer for patients experiencing homelessness (57 [14-96] vs 48 [0-81] days), with overlap in the IQRs (Table 3).

**Table 3.** Median Time From Diagnosis to Treatment, Stratified by Veteran Housing Status

Characteristic	Lung cancer			Colorectal cancer			Breast cancer		
	Time from diagnosis to treatment, median (IQR), d		*P* value^a^	Time from diagnosis to treatment, median (IQR), d		*P* value	Time from diagnosis to treatment, median (IQR), d		*P* value
	With housing	Without housing		With housing	Without housing		With housing	Without housing	
Start of chemotherapy	49 (28-82)	51 (27-85)	.28	62 (40-91)	64 (43-94)	.26	79 (50-112)	82 (59-116)	.36
Most definitive surgery	48 (0-81)	57 (14-96)	<.001	27 (0-58)	26 (0-69)	.84	48 (29-81)	50 (28-84)	.40
Start of radiation	57 (34-92)	59 (34-98)	.14	55 (38-85)	61 (41-86)	.048	135 (93-224)	162 (99-251)	.04

^a^ All *P* values are derived from Wilcoxon rank-sum tests.

### Surgical Outcomes

We included 25 737 operations in our analysis, 1225 (5%) of which were among patients experiencing homelessness. There was no difference in the proportion of surgical procedures captured in the VASQIP by housing status (eTable 1 in Supplement 1). Demographics of patients included in the analysis of surgical outcomes are displayed in eTable 2 in Supplement 1. There was no difference in the incidence of emergent operations, postoperative admissions, or any postoperative complications (including surgical site infection, urinary tract infection, myocardial infarction, stroke, or deep venous thrombosis) between patient groups by housing status. Patients experiencing homelessness had a higher rate of pulmonary emboli after lung cancer operation (1.3% vs 0.6%). In addition, patients experiencing homelessness had notably longer mean (SD) lengths of stay for all cancer types (lung: 8.2 [8.0] vs 7.4 [7.1] days; colorectal: 9.8 [11.7] vs 8.2 [7.5] days; and breast: 2.7 [2.4] vs 2.1 [3.1] days). These patients were also less likely to be discharged to the community in all cancer types studied (Table 4).

**Table 4.** Outcomes Following Definitive Oncologic Operation, Stratified by Veteran Housing Status^a^

Characteristic	Lung cancer (n = 11 224)			Colorectal cancer (n = 12 191)			Breast cancer (n = 332)		
	With housing (n = 10 700)	Without housing (n = 524)	*P* value	With housing (n = 11 601)	Without housing (n = 590)	*P* value	With housing (n = 2211)	Without housing (n = 111)	*P* value
Emergent case	11 (0.1)	2 (0.4)	.12	493 (4)	28 (5)	.53	2 (<0.1)	0	>.99
Inpatient admission status	9250 (86)	451 (86)	.79	9802 (84)	504 (85)	.60	795 (36)	46 (41)	.27
Length of stay, mean (SD), d	7.4 (7.1)	8.2 (8.0)	.03	8.2 (7.5)	9.8 (11.7)	<.001	2.1 (3.1)	2.7 (2.4)	.01
Disposition after surgery									
Community	10 100 (96)	473 (93)	.002	10 433 (93)	511 (90)	.04	1326 (98)	69 (91)	<.001
Nursing home	251 (2)	22 (4)		570 (5)	39 (7)		7 (1)	7 (9)	
Other	166 (2)	15 (3)		275 (2)	20 (4)		20 (2)	0	
Return to the operating room	552 (5)	23 (4)	.48	888 (8)	43 (7)	.81	179 (8)	11 (10)	.48
Any postoperative complication	1365 (13)	64 (12)	.79	2187 (19)	119 (20)	.42	82 (4)	5 (5)	.61
Wound disruption	17 (0.2)	0	>.99	200 (2)	5 (1)	.14	7 (0.3)	1 (1)	.32
Superficial surgical site infection	102 (1)	1 (0.2)	.09	544 (5)	30 (5)	.62	40 (2)	3 (3)	.46
Deep incisional surgical site infection	21 (0.2)	1 (0.2)	>.99	149 (1)	10 (2)	.35	12 (1)	0	>.99
Organ space surgical site infection	67 (1)	2 (0.4)	.77	337 (3)	22 (4)	.26	1 (<0.1)	0	>.99
Urinary tract infection	115 (1)	2 (0.4)	.18	237 (2)	15 (3)	.37	7 (0.3)	0	>.99
Myocardial infarction	44 (0.4)	0	.27	72 (1)	1 (0.2)	.27	0	0	
Stroke	38 (0.4)	0	.42	39 (0.3)	0	.26	1 (<0.1)	0	>.99
Deep vein thrombosis	50 (1)	1 (0.2)	.73	110 (1)	2 (0.3)	.18	2 (<0.1)	0	>.99
Pulmonary embolism	62 (1)	7 (1)	.04	102 (1)	2 (0.3)	.25	1 (<0.1)	1 (1)	.09

^a^ Unless indicated otherwise, data are presented as the No. (%) of patients. Percentages may not total 100 owing to rounding.

### Overall Survival

In the unadjusted survival curve for all-cause mortality for breast cancer, lower survival rates were observed for patients experiencing homelessness (eFigure in Supplement 1). Patients with lung or colorectal cancer and experiencing homelessness did not have statistically significantly different survival curves compared with patients with housing.

### Adjusted Survival

After adjusting for age at diagnosis, sex, stage at diagnosis, race, ethnicity, marital status, facility location, and comorbidities, patients experiencing homelessness had higher rates of mortality after 3 months for lung and colorectal rectal cancers, with adjusted hazard ratios of 1.1 (95% CI, 1.1-1.2) and 1.3 (95% CI, 1.2-1.4; both *P* < .001), respectively. The adjusted hazard ratio for mortality after 3 months for breast cancer was 1.2 (95% CI, 0.8-1.8; *P* = .27) (Table 5).

**Table 5.** Unadjusted and Adjusted Cox Proportional Hazards Regression Models for All-Cause Mortality^a^

Time after diagnosis, mo	Unadjusted model		Adjusted model^b^	
	HR (95% CI)	*P* value	HR (95% CI)	*P* value
Lung				
≤3	1.0 (0.9-1.0)	.33	1.0 (0.9-1.1)	.80
>3	1.1 (1.0-1.1)	.01	1.1 (1.1-1.2)	<.001
Colorectal				
≤3	0.7 (0.6-0.9)	.002	0.7 (0.6-1.0)	.02
>3	1.2 (1.1-1.3)	<.001	1.3 (1.2-1.4)	<.001
Breast				
≤3	1.2 (0.4-3.4)	.70	0.8 (0.2-3.3)	.76
>3	1.6 (1.2-2.1)	.01	1.2 (0.8-1.8)	.27

Abbreviation: HR, hazard ratio.

^a^ Patients who changed housing status after diagnosis were censored at the time of housing status change.

^b^ Adjusted for age at diagnosis, sex, stage at diagnosis, race, ethnicity, marital status, facility location, and mental or physical health comorbidities.

## Discussion

This study examined US veterans receiving VA care who were diagnosed with lung, colorectal, or breast cancer between 2011 and 2020. We observed that patients experiencing homelessness had 1.1 to 1.3 times the risk of mortality for lung and colorectal cancers beyond 3 months after diagnosis compared with patients with housing. We did not observe any statistically significant differences in adjusted hazard ratios for death in patients with breast cancer. These findings differ in magnitude from other research examining cancer outcomes in this population, which reported 1.7 to 2.3 times the rate of lung cancer mortality and 1.4 to 1.7 times the rate of colorectal cancer mortality in patients experiencing homelessness compared with patients with housing (eTable 3 in Supplement 1).^14,19^ We also observed that patients experiencing homelessness were more commonly diagnosed with metastatic colorectal cancer than patients with housing (22% vs 19%), but they were diagnosed at similar lung and breast cancer stages. Although this finding is consistent with other VA studies,^20^ it differs from other settings. For example, a study from the Boston Health Care for the Homeless Program reported that greater than 40% of colorectal cancers in patients experiencing homelessness were metastatic on presentation (compared with <20% of patients with housing) and almost 70% of breast cancers in these patients were locally advanced on presentation.^14^ Finally, we observed that veterans experiencing homelessness received similar cancer treatment to veterans with housing, with similar rates of loss to follow-up, similar overall times to treatment initiation, and similar rates of postoperative complications.

These more attenuated findings may be related to the unique strengths of the VA health system, which reduces financial barriers to care by providing health care to all members regardless of insurance status. We observed similar treatment patterns between patient groups regardless of housing status, which suggests that access to oncologic care may not be as large of a barrier for patients in the VA system experiencing homelessness compared with other settings. We saw some evidence of differences in care provided for patients experiencing homelessness, including being less likely to have more than 12 lymph nodes examined during surgery for colorectal cancer, waiting substantially longer for definitive surgery for lung cancer, and waiting longer to initiate radiation therapy for colorectal and breast cancers. However, these differences were less pronounced than in other settings, in which patients with cancer who were also experiencing homelessness more commonly had treatment delays,^21,22^ missed appointments,^21^ and incomplete treatment.^21^ However, there may be unmeasured differences in therapy completion and adherence to guideline-concordant care, which could contribute to the observed differences in outcomes. There may be many reasons for differential adherence to care guidelines at both the individual (patient and physician) and system levels, which ought to be further explored qualitatively.

Our findings related to stage at diagnosis may reflect improved access to primary care and screening in VA-treated patients experiencing homelessness compared with non-VA settings, particularly for lung and breast cancers. In our cohort, both patient groups had similar access to primary care in the year before diagnosis. However, a greater proportion of patients experiencing homelessness presented with metastatic colorectal cancer, which may be related to barriers in timely screening. Barriers described in other vulnerable groups, including lower rates of physician recommendation, limited health literacy, and distrust in the health care system, likely apply to patients experiencing homelessness as well.^23^ Additionally, other social and structural barriers unique to patients experiencing homelessness (including lack of private bathrooms and competing priorities) may be important to address as well.^24,25^

When investigating outcomes after cancer surgery, we observed longer lengths of stay for individuals experiencing homelessness. However, this difference was smaller than observed postsurgically in other settings,^26^ which may be related to supportive discharge options available to patients experiencing homelessness cared for at the VA. In our study, a higher proportion of patients experiencing homelessness were discharged to noncommunity settings, which is consistent with other surgical literature at the VA.^27^

The VA has made addressing veteran homelessness a priority since 2009^28^ by investing in dedicated housing resources to meet veteran needs, including homelessness prevention, rapid rehousing, rental subsidies, and permanent supportive housing.^29^ This commitment has resulted in a 45% reduction in counts of veterans experiencing homelessness on a single night between 2009 and 2017 (compared with much smaller reductions in the overall US population experiencing homelessness).^30,31^ In our cohort, we observed that almost 20% of veterans experiencing homelessness gained housing after cancer diagnosis. Further research should be undertaken to evaluate whether providing housing after cancer diagnosis helps improve outcomes among patients experiencing homelessness.^21,22,23,24,25^

Of note, in this study, a markedly higher proportion of individuals experiencing homelessness were Black compared with individuals with housing (37% vs 17%). This difference is consistent with the overall demographics of individuals experiencing homelessness in the US, with Black individuals overrepresented due to structural racism.^32^ Given that there are well-documented racial disparities in cancer care and oncologic outcomes, there may have been interaction between race and housing status that was associated with overall outcomes, which should be explored in further studies.^33^

Finally, we observed that veterans experiencing homelessness were diagnosed at younger ages (by 4-7 years) for all cancers studied. This finding aligns with other literature highlighting earlier onset of chronic diseases in populations experiencing homelessness,^34^ and it may prompt clinicians to have a higher index of suspicion for malignant neoplasm in younger patients with appropriate symptoms who are experiencing homelessness.

Many factors contribute to the likelihood of survival after a cancer diagnosis, including biological, environmental, behavioral, health care, economic, political, and social determinants.^35^ Lack of housing is a critical factor that interacts across many of these domains with consequences on health outcomes—ranging from poor access to ambulatory care,^36^ to bias and stigma, to difficulty navigating complex health care systems due to competing priorities,^37^ and to vulnerability to external forces such as law enforcement or shelter requirements.^38^ However, this individual-level social determinant of health has been understudied, particularly in cancer, in part because of the lack of systematic documentation of housing status. The VA has one of the most robust documentation systems of homelessness in the country, due to multiple different layers of inquiry and reporting in both the inpatient and outpatient settings.^39^ This contrasts with other administrative mechanisms of reporting housing, which rely primarily on zip codes and *International Classification of Diseases, Tenth Revision, Clinical Modification* Z codes, both of which have been shown to be markedly underused.^40^ A more nuanced understanding of the consequences of housing status on cancer care and outcomes will only be possible with more complete documentation in other health systems.

### Limitations

This study has certain limitations. Our sample only included veterans using VA health care. Therefore, the results may not be generalizable to other veterans experiencing homelessness who are not in the VA system and to nonveteran populations experiencing homelessness. Depending on the volume and expertise of the center, veterans may seek community care for certain cancers (particularly breast cancer). However, we sought to understand the cancer outcomes and care experiences of veterans cared for in the VA system. Our evaluation of surgical outcomes is limited to only a subset of procedures performed at the VA. However, the VASQIP tends to report more complex surgeries, especially those that require inpatient admission, so we anticipate that many of the complications in both groups are accounted for in our analysis.^35,36,37,38,39,40^

## Conclusions

The findings of this cohort study suggest that differences in oncologic outcomes in breast, colorectal, and lung cancers between VA patients experiencing homelessness and those with housing are present but small. This result differs from findings in other settings. There may be important lessons to learn from the VA that other health systems can adopt to improve oncologic outcomes for patients experiencing homelessness.

## Notes

Supplement 1. **eAppendix.** Determination of Housing Status in US Department of Veterans Affairs Health System Data **eTable 1.** Proportion of All Procedures Identified in the Veterans Affairs Surgical Quality Improvement Program (VASQIP) **eTable 2.** Demographics of Patients Included in Surgical Outcomes Analysis **eFigure.** Kaplan-Meier Curves for All-Cause Mortality for Lung, Colorectal, and Breast Cancer, Stratified by Housing Status **eTable 3.** Comparison of Mortality Differences Between Unhoused and Housed Patients With Cancer in Other Studies **eTable 4.** Demographic Characteristics of Veterans Diagnosed With Lung, Colorectal, and Breast Cancer Stratified by Housing Status

Supplement 2.
Data Sharing Statement
