# Supplementary material for: Housing Status, Cancer Care, and Associated Outcomes Among US Veterans
Source: JAMA Netw Open. 2023 Dec 21;6(12):e2349143. doi: 10.1001/jamanetworkopen.2023.49143 (PMC10739065; doi:10.1001/jamanetworkopen.2023.49143)
Supplement: Supplement 1. — eAppendix. Determination of Housing Status in US Department of Veterans Affairs Health System Data eTable 1. Proportion of All Procedures Identified in the Veterans Affairs Surgical Quality Improvement Program (VASQIP) eTable 2. Demographics of Patients Included in Surgical Outcomes Analysis eFigure. Kaplan-Meier Curves for All-Cause Mortality for Lung, Colorectal, and Breast Cancer, Stratified by Housing Status eTable 3. Comparison of Mortality Differences Between Unhoused and Housed Patients With Cancer in Other Studies eTable 4. Demographic Characteristics of Veterans Diagnosed With Lung, Colorectal, and Breast Cancer Stratified by Housing Status [file jamanetwopen-e2349143-s001.pdf]

Supplementary Online Content

Decker HC, Graham LA, Titan A, et al. Housing status, cancer care, and associated outcomes among US veterans. *JAMA Netw Open*. 2023;6(12):e2349143. doi:10.1001/jamanetworkopen.2023.49143

eAppendix. Determination of Housing Status in US Department of Veterans Affairs Health System Data

eTable 1. Proportion of All Procedures Identified in the Veterans Affairs Surgical Quality Improvement Program (VASQIP)

eTable 2. Demographics of Patients Included in Surgical Outcomes Analysis

eFigure. Kaplan-Meier Curves for All-Cause Mortality for Lung, Colorectal, and Breast Cancer, Stratified by Housing Status

eTable 3. Comparison of Mortality Differences Between Unhoused and Housed Patients With Cancer in Other Studies

eTable 4. Demographic Characteristics of Veterans Diagnosed With Lung, Colorectal, and Breast Cancer Stratified by Housing Status

This supplementary material has been provided by the authors to give readers additional information about their work.

© 2023 Decker HC et al. *JAMA Network Open.*

eAppendix. Determination of Housing Status in US Department of Veterans Affairs Health System Data

Outpatient stop codes	501	Homeless mentally ill outreach	% Unhoused Identified*
	504	Grant and Per Diem group	85.6%
	507	Housing and Urban Development-Veterans Affairs Supportive Housing group	
	508	Healthcare for Homeless Veterans Group	
	511	Grant and Per Diem Group	
	522	Housing and Urban Development-Veterans Affairs Supportive Housing group	
	528	Telephone Mental Health Intensive Case Management	
	529	Healthcare for Homeless Veterans Group	
	530	Telephone: Housing and Urban Development-Veterans Affairs Supportive Housing group	
	590	Community outreach homeless	
Clinical Reminder s		Homeless Screener Clinical Reminder	83.1%
Inpatient specialty IENs	28	Homeless Compensated Work Therapy/ Transitional Residence	0.03%
	29	Substance abuse Compensated Work Therapy/ Transitional Residence	
	37	Domiciliary Care for Homeless Veterans	
	39	General Compensated Work Therapy/ Transitional Residence	
ICD-9	V60.0	Lack of housing	52.9%

© 2023 Decker HC et al. *JAMA Network Open.*

ICD-10	Z59.0	Homelessness	
Homeless Operations Management Evaluation System (HOMES)	CERS	Contracted Emergency Residential Services	33.9%
	CWT/TR	Compensated Work Therapy / Transitional Residence	
	DCHV	Domiciliary Care for Homeless Veterans	
	GPD	Grant and Per Diem	
	HCHV	Health Care for Homeless Veterans	
	HUD-VASH	HUD-VA Supportive Housing	
	LDSH	Low Demand Safe Haven	
HMIS	SSVF	Supportive Services for Veteran Families	

*The categories are not mutually exclusive and may not sum to 100% as Veterans can be identified via multiple sources

© 2023 Decker HC et al. *JAMA Network Open.*

eTable 1. Proportion of All Procedures Identified in the Veterans Affairs Surgical Quality Improvement Program (VASQIP)

**Proportion of All Procedures Identified in VASQIP**				
	**Overall**	**Housed**	**Homeless**	**P value**
**Lung**	12,272 (41.5%)	11,642 (41.5%)	630 (41.5%)	0.99
**Colorectal**	11,727 (49.0%)	11,099 (49.1%)	628 (48.4%)	0.65
**Breast**	2,176 (37.4%)	2,058 (37.4%)	118 (27.0%)	0.88

© 2023 Decker HC et al. *JAMA Network Open.*

eTable 2. Demographics of Patients Included in Surgical Outcomes Analysis

	**Lung**			**Colorectal**			**Breast**		
	**Housed**, N = 10,700	**Unhoused**, N = 524	**p-value**[1]	**Housed**, N = 11,601	**Unhoused**, N = 590	**p-value**[1]	**Housed**, N = 2,211	**Unhoused**, N = 111	**p-value**[1]
**Sex, n (%)**			0.1			0.32			0.8
Female	450 (4.2)	30 (5.7)		437 (3.8)	17 (2.9)		1,829 (83)	91 (82)	
Male	10,250 (96)	494 (94)		11,164 (96)	573 (97)		382 (17)	20 (18)	
**Race, n (%)**			**<0.001**			**<0.001**			**<0.001**
American Indian	51 (0.5)	1 (0.2)		96 (0.9)	8 (1.5)		24 (1.2)	3 (3.0)	
Asian	19 (0.2)	2 (0.4)		50 (0.5)	4 (0.8)		19 (1.0)	0 (0)	
Black	1,457 (15)	208 (43)		1,941 (19)	216 (41)		582 (30)	49 (49)	
White	8,107 (84)	271 (56)		8,213 (80)	305 (57)		1,307 (68)	48 (48)	
Missing	1,066	42		1,301	57		279	11	
**Age, years**			**<0.001**			**<0.001**			**<0.001**
Mean (SD)	68.03 (6.98)	62.72 (6.68)		68.06 (10.32)	61.53 (9.22)		59.50 (11.45)	55.23 (9.03)	
**BMI**			**<0.001**			**<0.001**			0.32
Mean (SD)	27.39 (5.59)	25.93 (5.23)		28.51 (6.26)	27.29 (6.87)		32.26 (88.69)	30.78 (6.38)	
**ASA Classification, n (%)**			**0.029**			0.26			0.32
1	2 (<0.1)	0 (0)		33 (0.3)	1 (0.2)		27 (1.2)	0 (0)	
2	257 (2.4)	24 (4.6)		1,400 (12)	56 (9.5)		681 (31)	27 (24)	
3	8,859 (83)	433 (83)		8,656 (75)	451 (76)		1,441 (65)	82 (74)	
4	1,580 (15)	67 (13)		1,489 (13)	80 (14)		62 (2.8)	2 (1.8)	
5	1 (<0.1)	0 (0)		21 (0.2)	2 (0.3)				
**Functional Status, n (%)**			0.49			0.98			>0.99
Independent	10,453 (98)	510 (97)		10,923 (94)	556 (94)		2,154 (97)	109 (98)	
Partially Dependent	234 (2.2)	13 (2.5)		587 (5.1)	30 (5.1)		48 (2.2)	2 (1.8)	
Totally Dependent	12 (0.1)	1 (0.2)		90 (0.8)	4 (0.7)		9 (0.4)	0 (0)	

© 2023 Decker HC et al. *JAMA Network Open.*

**Diabetes, n (%)**			**<0.001**			**<0.001**			0.46
None	8,168 (76)	455 (87)		8,258 (71)	451 (76)		1,772 (80)	89 (80)	
Diet Only	1,554 (15)	37 (7.1)		1,920 (17)	66 (11)		260 (12)	16 (14)	
Requires Insulin	978 (9.1)	32 (6.1)		1,423 (12)	73 (12)		179 (8.1)	6 (5.4)	
**History of COPD, n (%)**	4,857 (45)	234 (45)	0.75	1,709 (15)	93 (16)	0.48	177 (8.0)	11 (9.9)	0.47
**History of MI, n (%)**	700 (6.5)	18 (3.4)	**0.003**	638 (5.5)	32 (5.4)	>0.99	59 (2.7)	0 (0)	0.11
**History of CHF, n (%)**	1,434 (14)	40 (7.8)	**<0.001**	1,293 (11)	59 (10)	0.42	107 (4.9)	4 (3.7)	0.82
**Previous Cardiac Surgery, n (%)**	892 (8.4)	22 (4.2)	**<0.001**	937 (8.1)	34 (5.8)	**0.043**	61 (2.8)	1 (0.9)	0.37
**Hypertension Requiring Medication, n (%)**	7,907 (74)	330 (63)	**<0.001**	8,152 (70)	381 (65)	**0.004**	1,187 (54)	69 (62)	0.1
**History of PVD, n (%)**	684 (6.4)	21 (4.0)	**0.027**	283 (2.4)	16 (2.7)	0.68	11 (0.5)	2 (1.8)	0.13
**Impaired Sensorium, n (%)**	8 (<0.1)	2 (0.4)	0.076	28 (0.2)	1 (0.2)	>0.99	1 (<0.1)	0 (0)	>0.99
**History of TIA, n (%)**	365 (3.4)	12 (2.3)	0.21	290 (2.5)	12 (2.0)	0.59	56 (2.5)	1 (0.9)	0.52
**CVA/Stroke with no neurological deficit, n (%)**	446 (4.2)	17 (3.2)	0.37	394 (3.4)	18 (3.1)	0.73	40 (1.8)	2 (1.8)	>0.99
**CVA/Stroke with neurological deficit, n (%)**	329 (3.1)	20 (3.8)	0.36	389 (3.4)	20 (3.4)	0.91	53 (2.4)	1 (0.9)	0.52

© 2023 Decker HC et al. *JAMA Network Open.*

eFigure. Kaplan-Meier Curves for All-Cause Mortality for Lung, Colorectal, and Breast Cancer, Stratified by Housing Status

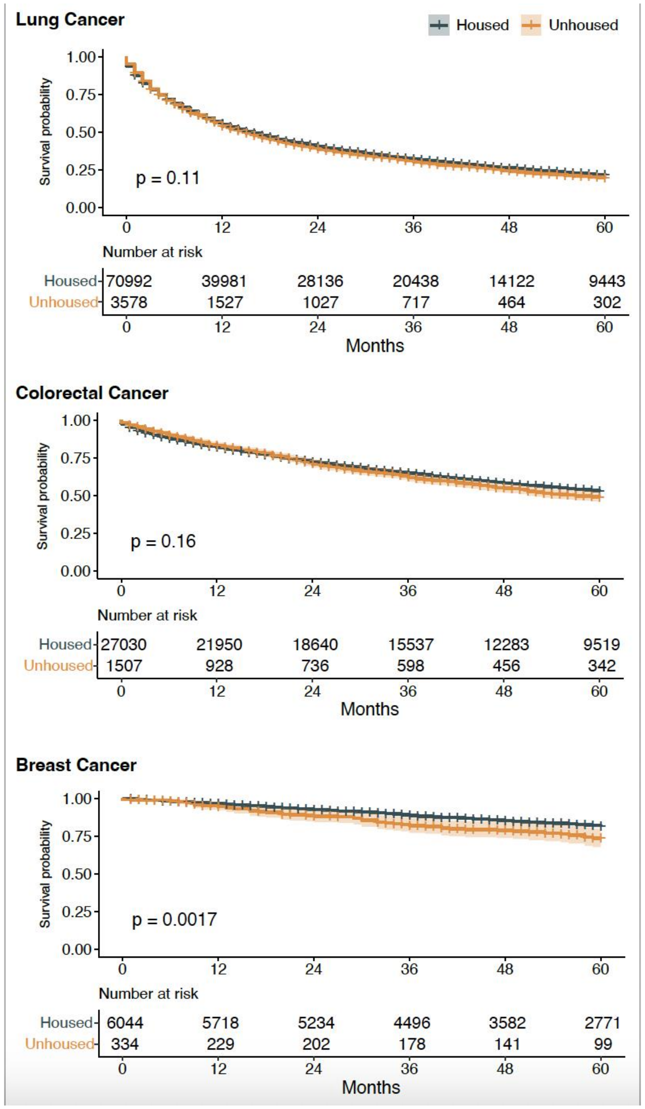

© 2023 Decker HC et al. *JAMA Network Open.*

eTable 3. Comparison of Mortality Differences Between Unhoused and Housed Patients With Cancer in Other Studies

Study	Geographic Location	Setting	Measure Reported	All Sites (Measure, 95% CI)	Lung	Colorectal	Breast
Baggett et al, 2016	Boston, United States	Healthcare for the Homeless Program	Standardized Mortality Ratios	1.61 (1.14–2.20)	2.31 (1.26–3.88)	1.61 (0.33–4.72)	1.07 (0.35–2.50)
Hwang et al, 2009	Canada	Nationwide	Mortality Rate Ratios	Men: 1.56 (1.44 - 1.70)<br><br>Women:1.38 (1.18 - 1.62)	Men: 1.91 (1.67 - 2.18)<br><br>Women: 1.73 (1.26 - 2.36)	Men: 1.39 (1.08 - 1.78)<br><br>Women: 1.67 (1.12 - 2.51)	Men: Not reported<br><br>Women: 1.58 (1.11 - 2.24)

© 2023 Decker HC et al. *JAMA Network Open.*

eTable 4. Demographic Characteristics of Veterans Diagnosed With Lung, Colorectal, and Breast Cancer Stratified by Housing Status (Supplement to Table 1)

	Overall			Lung			Colorectal			Breast		
	**Housed**, N = 104,129	**Unhoused**, N = 5,356	**p-value**	Housed, N = 71,036	Unhoused, N = 3,534	p-value	Housed, N = 27,040	Unhoused, N = 1,497	p-value	Housed, N = 6,053	Unhoused, N = 325	p-value
**BMI at Diagnosis, Mean (SD)**	27.4 (6.2)	26.1 (6.2)	**<0.001**	26.6 (5.9)	25.1 (5.7)	**<0.001**	28.6 (6.2)	27.3 (6.3)	**<0.001**	31.0 (7.0)	31.2 (7.5)	0.66
**Charlson Comorbidity Index, Mean (SD)**			0.90	3.6 (3.2)	3.7 (3.4)	0.93	2.7 (3.0)	2.9 (3.2)	0.43	1.3 (1.9)	1.5 (2.1)	0.09
**Diabetes with complications, n (%)**	13,418 (13)	585 (11)	**<0.001**	9,140 (13)	384 (11)	**<0.001**	3,827 (14)	179 (12)	0.018	451 (7.5)	22 (6.8)	0.74
**Depression, Bipolar, or Other Mood Disorders, n (%)**	17,974 (17)	2,009 (38)	**<0.001**	12,342 (17)	1,358 (38)	**<0.001**	3,898 (14)	478 (32)	**<0.001**	1,734 (29)	173 (53)	**<0.001**
**Dementia, n (%)**	2,672 (2.6)	135 (2.5)	0.89	1,872 (2.6)	82 (2.3)	0.28	729 (2.7)	48 (3.2)	0.25	71 (1.2)	5 (1.5)	0.59
**Reported Tobacco Use, n (%)**			**<0.001**			**<0.001**			**<0.001**			**<0.001**
No	12,742 (13)	480 (9.2)		2,431 (3.5)	75 (2.2)		7,506 (29)	289 (20)		2,805 (49)	116 (38)	
Current	46,499 (46)	3,483 (67)		37,506 (54)	2,618 (76)		7,650 (30)	743 (52)		1,343 (23)	122 (40)	
Prior	41,480 (41)	1,227 (24)		29,524 (43)	772 (22)		10,355 (41)	385 (27)		1,601 (28)	70 (23)	
**Reported Alcohol Use, n (%)**			**<0.001**			**<0.001**			**<0.001**			**0.01**
No	35,276 (38)	1,326 (27)		22,536 (35)	783 (24)		9,846 (40)	399 (29)		2,894 (52)	144 (48)	
Current	37,812 (40)	2,320 (47)		25,892 (41)	1,551 (48)		9,804 (40)	660 (48)		2,116 (38)	109 (37)	
Prior	20,829 (22)	1,253 (26)		15,452 (24)	894 (28)		4,867 (20)	314 (23)		510 (9.2)	45 (15)	
**Myocardial Infarction, n (%)**	5,549 (5.3)	297 (5.5)	0.49	4,268 (6.0)	219 (6.2)	0.64	1,185 (4.4)	74 (4.9)	0.30	96 (1.6)	4 (1.2)	0.82
**Congestive Heart Failure, n (%)**	13,035 (13)	672 (13)	0.95	9,829 (14)	488 (14)	0.98	2,946 (11)	171 (11)	0.52	260 (4.3)	13 (4.0)	0.89
**Peripheral Vascular Disease, n (%)**	20,969 (20)	822 (15)	**<0.001**	17,237 (24)	649 (18)	**<0.001**	3,499 (13)	155 (10)	**0.003**	233 (3.8)	18 (5.5)	0.14
**Cerebrovascular Disease, n (%)**	13,927 (13)	563 (11)	**<0.001**	10,795 (15)	422 (12)	**<0.001**	2,819 (10)	121 (8.1)	**0.003**	313 (5.2)	20 (6.2)	0.44
**Chronic Pulmonary Disease, n (%)**	47,546 (46)	2,443 (46)	0.96	40,177 (57)	2,005 (57)	0.85	6,224 (23)	368 (25)	0.17	1,145 (19)	70 (22)	0.25
**Mild Liver Disease, n (%)**	8,756 (8.4)	972 (18)	**<0.001**	6,367 (9.0)	700 (20)	**<0.001**	2,115 (7.8)	244 (16)	**<0.001**	274 (4.5)	28 (8.6)	**0.002**
**Moderate or Severe Liver Disease, n (%)**	901 (0.9)	88 (1.6)	**<0.001**	629 (0.9)	57 (1.6)	**<0.001**	253 (0.9)	29 (1.9)	**<0.001**	19 (0.3)	2 (0.6)	0.29

© 2023 Decker HC et al. *JAMA Network Open.*
