# Supplementary material for: Housing Status, Cancer Care, and Associated Outcomes Among US Veterans
Source: JAMA Netw Open. 2023 Dec 21;6(12):e2349143. doi: 10.1001/jamanetworkopen.2023.49143 (PMC10739065; doi:10.1001/jamanetworkopen.2023.49143)
Supplement: Supplement 2. — Data Sharing Statement [file jamanetwopen-e2349143-s002.pdf]

Data Sharing Statement

Decker. Housing Status, Cancer Care, and Associated Outcomes Among US Veterans. *JAMA Netw Open*. Published December 21, 2023. doi:10.1001/jamanetworkopen.2023.49143

Data
Data available: No

Additional Information
Explanation for why data not available: This data includes sensitive housing data about US veterans (a protected population). However, we will offer detailed description of how we did the analysis (including code for our exposure and outcomes) for other researchers with approved access to VA data.
